# Supplementary material for: Effects of segmentation errors on downstream-analysis in highly-multiplexed tissue imaging
Source: PLoS Comput Biol. 2025 Sep 15;21(9):e1013350. doi: 10.1371/journal.pcbi.1013350 (PMC12456762; doi:10.1371/journal.pcbi.1013350)
Supplement: S1 Text — (PDF) [file pcbi.1013350.s001.pdf]

F1	Rotation	Scaling	Shearing	Translation
70	(-1, 1)	(0.93, 1.04)	(0, 0)	(-7, 7)
80	(-6, 6)	(0.87, 1.1)	(-6, 6)	(-6, 6)
90	(-5, 5)	(0.88, 1.08)	(-5, 5)	(-5, 5)
100	(-1, 1)	(0.8, 1.14)	(-1, 1)	(-1, 1)
