# Supplementary figures and images for: Effects of segmentation errors on downstream-analysis in highly-multiplexed tissue imaging
Source: PLoS Comput Biol. 2025 Sep 15;21(9):e1013350. doi: 10.1371/journal.pcbi.1013350 (PMC12456762; doi:10.1371/journal.pcbi.1013350)

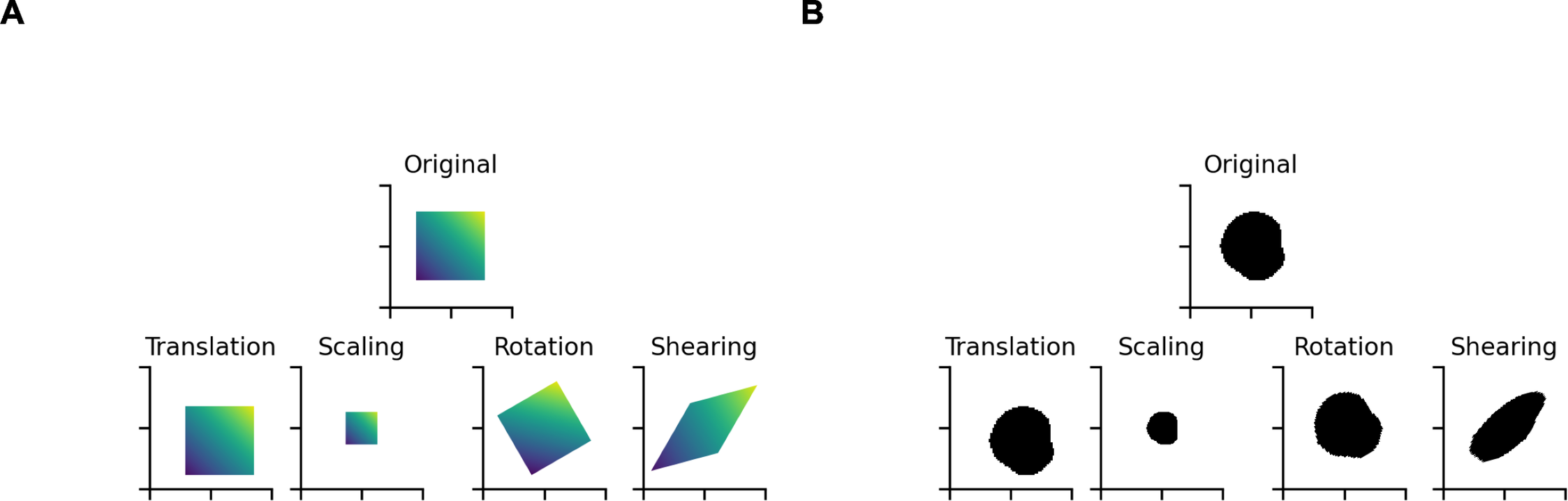

Supplement: S1 Fig — (A) Minimal example showcasing the effect of single affine transformations on a simple shape. (B) The same transformations applied to a single cell segmentation mask. (TIF) [file pcbi.1013350.s002.tif]

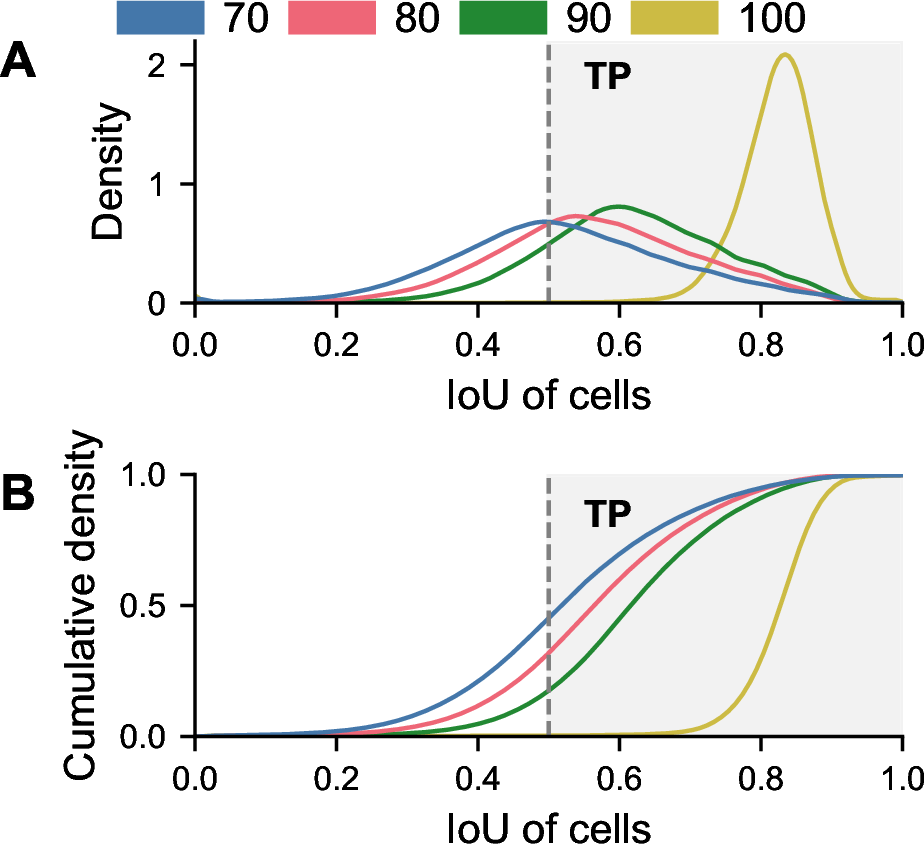

Supplement: S2 Fig — (A) Histogram over IoU values of cells. (B) Cumulative histogram. Any value above 0.5 will be considered a true positive (TP). (TIF) [file pcbi.1013350.s003.tif]

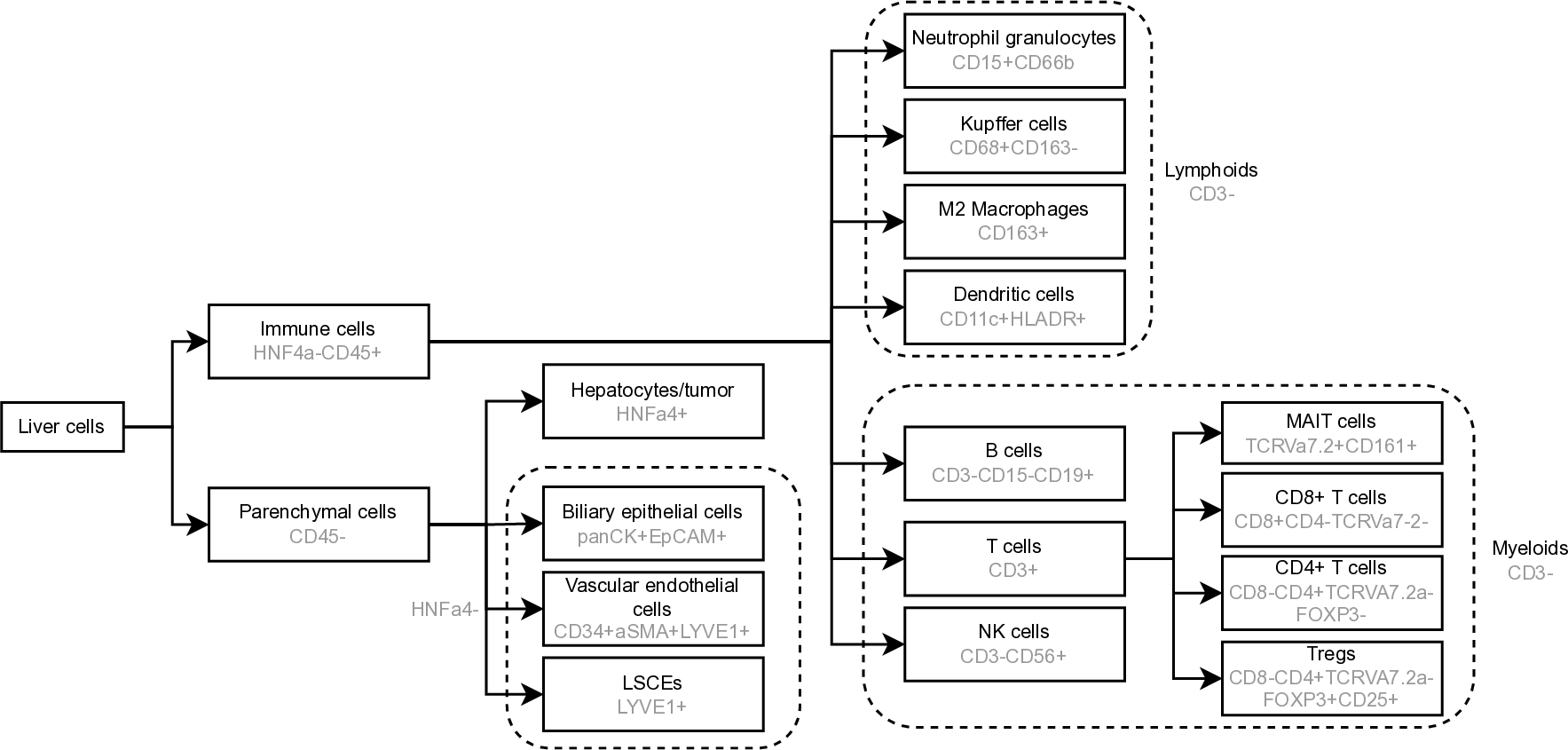

Supplement: S3 Fig — Myeloids and lymphocytes can not be assigned but are shown for visual reasons. (TIF) [file pcbi.1013350.s004.tif]

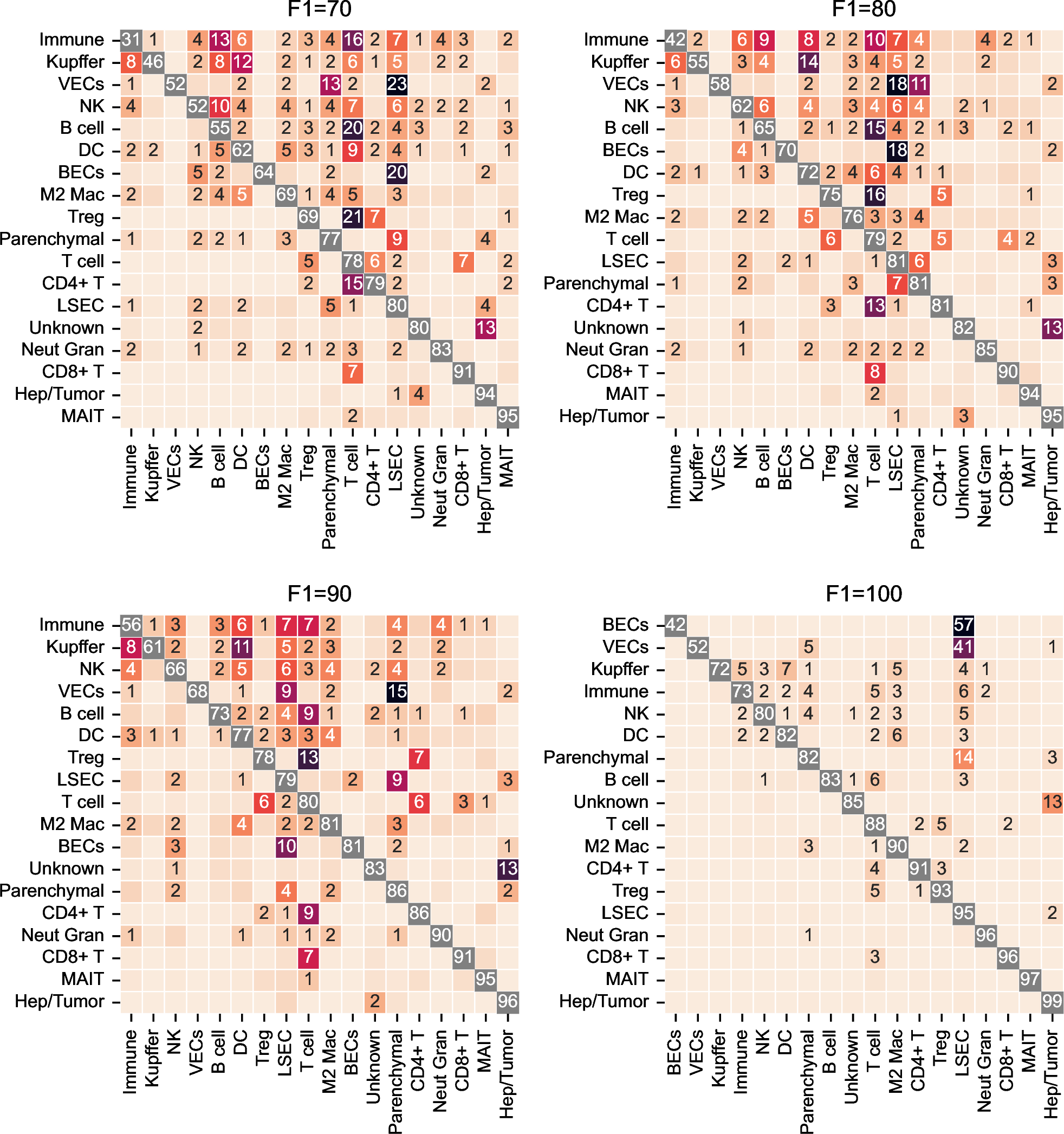

Supplement: S4 Fig — Only values ≥ 1 are shown. Color encodes the percentage of classified cells. For visual appearance, only off-diagonal elements are colored in. (TIF) [file pcbi.1013350.s005.tif]

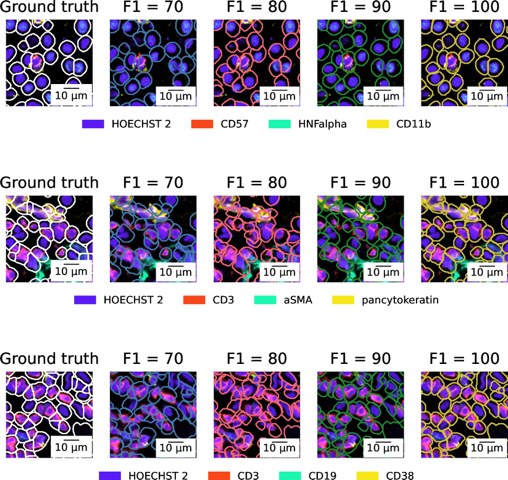

Supplement: S5 Fig — Each row shows a different tile, colors encode different marker expressions. The color of the mask outlines corresponds to perturbation strength. Empty masks and merged masks are filtered in later QC steps. (TIF) [file pcbi.1013350.s006.tiff]

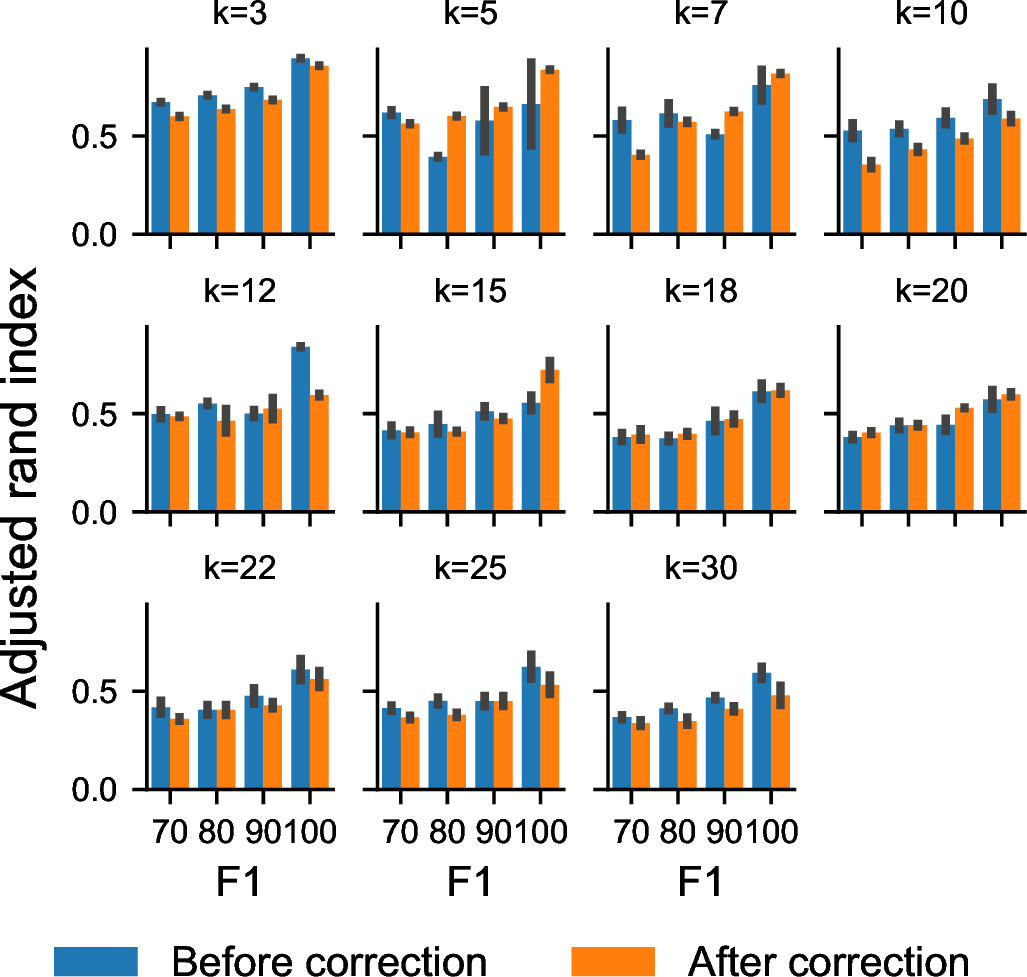

Supplement: S6 Fig — Each subplot shows the adjusted rand index for the initial k-Means clustering and after correction with STARLING. Error bars indicate one median absolute deviation. (TIF) [file pcbi.1013350.s007.tif]
