# Supplementary material for: Effects of segmentation errors on downstream-analysis in highly-multiplexed tissue imaging
Source: PLoS Comput Biol. 2025 Sep 15;21(9):e1013350. doi: 10.1371/journal.pcbi.1013350 (PMC12456762; doi:10.1371/journal.pcbi.1013350)

**Reviewers' comments:**

Reviewer's Responses to Questions

**Comments to the Authors:**
**Please note here if the review is uploaded as an attachment.**

Reviewer #1: This study provides a rigorous quantification of segmentation errors and their downstream effects, offering a much-needed benchmark for understanding error propagation in single-cell analyses. The use of affine transformations to simulate realistic errors is innovative, allowing systematic evaluation of segmentation robustness. The integration of k-Means and Leiden clustering alongside Gaussian Mixture Models broadens the findings interest and clear visualizations (e.g., Figures 2, 5, 6) make complex results interpretable. The study's practical implications extend to real-world spatial proteomics pipelines, emphasizing the necessity of improved segmentation evaluation metrics and quality control methods.

However, the authors reference a number of software tools that have previously been used to address the same or similar problems, including **STARLING, CyLinter, and ESQmodel**. This reviewer feels that the datasets generated by the authors could be run through those software to strengthen the findings in this manuscript and possibly to begin addressing some of the field issues the authors have noted.

Major Revisions

**Probabilistic Clustering with STARLING. The authors could compare standard clustering approaches (Leiden, k-Means) with STARLING, a segmentation-aware probabilistic model.** This could be done by running STARLING on the perturbed datasets and comparing the Adjusted Rand Index (ARI) and clustering stability against the current results (Figure 5). I**f STARLING improves clustering accuracy under segmentation errors, this would strengthen the study's impact.**

We thank the reviewer for the suggestion to evaluate STARLING, as a segmentation-aware probabilistic clustering model, against the currently considered clustering approaches.

Following this recommendation, we conducted a preliminary investigation by applying STARLING to our perturbed datasets. We then compared its clustering performance with the results obtained from our k-Means experiment.

Our initial findings from this exploratory analysis were inconclusive. We ran similar experiments as described for k-Means clustering. As per instructions in the STARLING documentation we only considered data that has been preprocessed with log(x+1). We used k-Means to generate the initial clustering.

As shown in Figure NUMBER, the clustering performance before and after correction depends on the selected number of clusters. Overall, STARLING shows the most consistent better performance for k=15 to k=20. In other cases (k=5, k=7) the results are mixed depending on the perturbation strength. For the remaining cases, the initial clustering agreement seems to be consistently better.

Although STARLING presents an intriguing methodology and its application to our data could be a subject for future exploration, we consider a comprehensive comparative analysis incorporating these mixed results to be beyond the defined scope of the current manuscript. We have therefore chosen to maintain our focus on the analyses conducted with the established clustering protocols as presented in the paper.

We added the generated results to the supplement and mentioned them accordingly in line 322 of the results section.

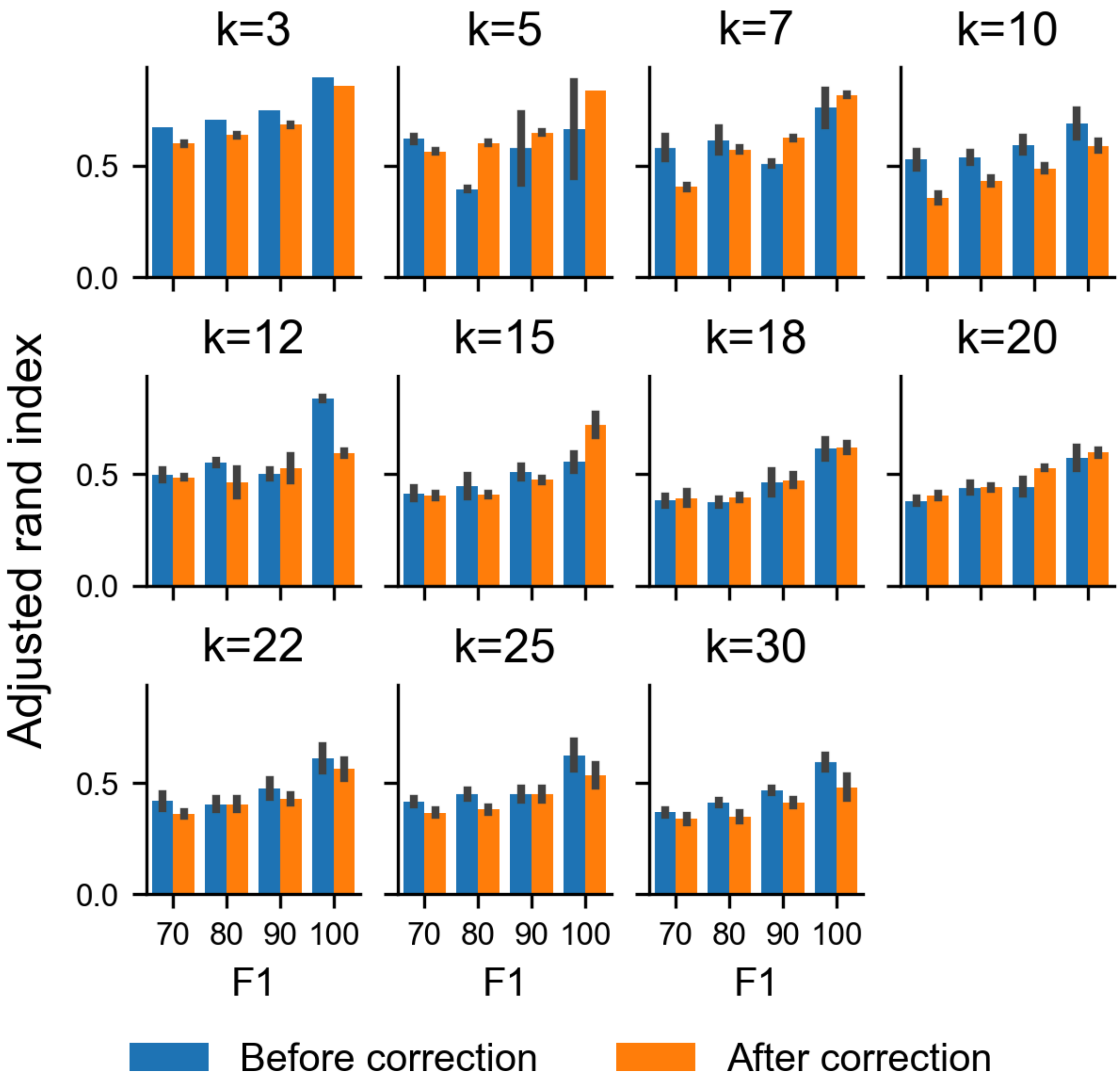


**Quality Control with CyLinter.** To assess the impact of quality control on segmentation artifacts, the authors could apply CyLinter to their perturbed datasets and remove flagged segmentations. By comparing clustering and phenotyping accuracy before and after artifact removal, they can quantify CyLinter's benefit (Figure 6B). This would demonstrate whether quality control can mitigate the negative

effects of segmentation errors.

We thank the reviewer for considering evaluation of CyLinter for assessing the impact of quality control on segmentation artifacts.

CyLinter comprises three primary modules for artifact removal: filtering based on expression intensity, cell size, and the correlation of imaging cycles. Our current data processing pipeline already incorporates rigorous quality control measures analogous to the first two of these modules (i.e., filtering by expression intensity and cell size, see Methods section line 64-65 "*Additionally, we perform quality control regarding cell size and cell nucleus signal (HOECHST) to remove empty cell masks and incorrectly merged cells.*"). These steps are integral to our workflow and are applied to the data presented.

The third module, concerning inter-cycle correlation, is unfortunately not applicable to our dataset due to the unavailability of the separate image cycles required for such an analysis. The available data consists of one aligned and stitched image per channel.

Given that our existing methodology already embeds key quality control principles similar to those in CyLinter, and the remaining component is inapplicable to our data, performing an additional comparative analysis as suggested would largely replicate our current practices or rely on unavailable data.

**Alternative Segmentation Evaluation with ESQmodel.** The manuscript critiques IoU-F1 thresholding but does not provide an alternative. The authors could apply ESQmodel, which evaluates segmentation accuracy with biologically informed metrics. By correlating ESQmodel scores with phenotyping errors (Figure 6) and clustering robustness (Figure 5), they can determine if it provides a more meaningful segmentation quality metric than IoU-F1.

We appreciate the reviewer's suggestion to explore ESQmodel as an alternative segmentation evaluation metric.

Upon investigation, we identified computational demands that preclude its application in our current study:

The implementation of ESQmodel results in a prohibitive computational cost for our dataset. We estimate that processing even a single one of our 80 generated perturbations would require over 92 hours, rendering a comprehensive analysis impractical. We arrived at this number by running the tool with different smaller datasets. As you can see in the figure presented below, the runtime scales linearly with the size of the analysed sample. We suspect this problem arises due to a) the choice of an inefficient sampling scheme (MCMC) and b) the chosen implementation of the aforementioned method.

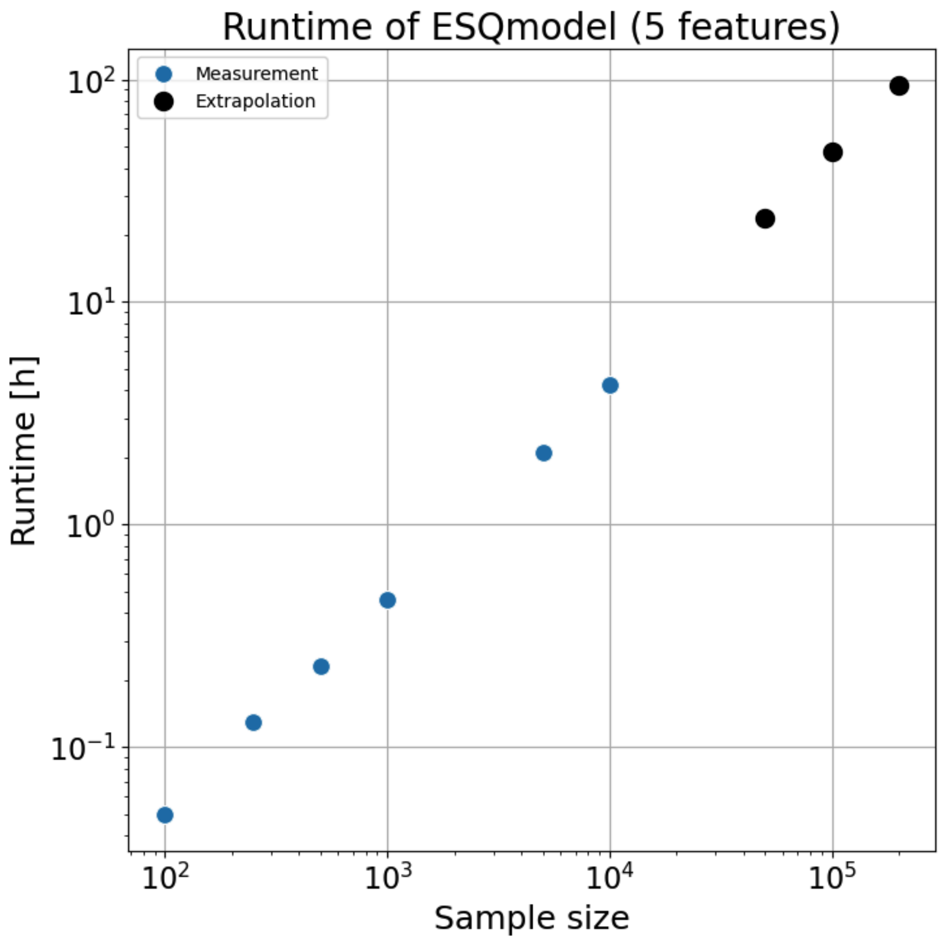


We acknowledge the merit of biologically informed metrics of the ESQmodel. However, we conclude that this model is an inapplicable option for our specific study due to the incompatibility of the computational demands in the context of the assessment of eighty perturbation variants of the original datasets.

Further, we consider our discussion of the IoU-F1 thresholding not as a fundamental critique of the metric. Instead we aim at pointing to and making the community aware of the effect of user-defined threshold on the dynamic range of the metric.

## Minor Revisions

**Scalability Considerations with MCMICRO.** The authors should discuss how MCMICRO could enable large-scale segmentation benchmarking. Addressing its modularity and efficiency in processing high-dimensional imaging data would clarify the study's applicability to larger datasets.

We thank the reviewer for this suggestion. As contributors to MCMICRO, we affirm its power for scalable image processing and executing segmentation pipelines, which are vital for large-scale benchmarking.

Our current study, however, concentrates on an in-silico evaluation methodology for segmentation robustness. While MCMICRO excels at scaling the segmentation pipelines that our framework evaluates, it is not primarily designed to orchestrate the in-silico experimental design and comparative analyses that form our core contribution. We added the following paragraph to the discussion section (line 416-420):

*As a broader framework, Nextflow is a suitable approach for these kinds of studies. By designing the full analysis as a Netflow pipeline, each part of it can be implemented as a module which allows for specific configuration. A similar approach has been applied by Zappia et al. to benchmark and compare feature selection for scRNA-seq data.*

**Probabilistic Correction for Phenotyping Errors.** The authors should propose a probabilistic post-processing correction for phenotyping errors, similar to STARLING's segmentation-aware approach. A Bayesian reassignment model could improve phenotype consistency, mitigating segmentation-induced misclassifications (Figure 6).

We thank the reviewer for this insightful suggestion regarding a probabilistic post-processing correction for phenotyping errors.

Developing and validating such a model is a potentially valuable direction. We are currently engaged in separate research focused on a novel probabilistic method for clustering, and the scope of this ongoing work already suggests it will merit its own dedicated publication.

Given that a robust probabilistic correction for phenotyping errors would similarly require significant methodological development and validation, we consider this an important but distinct avenue for future investigation, rather than an extension of the present study.

**Reviewer #2:** The authors demonstrate the effect of varying degrees of missegmentation on cell type calling in high plex tissues. The analysis is replete with comparisons of IOU and F1 scores and cell type clusterings. The intended audience appears to be computational biologists, image analysts, and data scientists. However, it might be challenging for an experimentalist to still fully appreciate the problem addressed in this paper. The data is present in the paper but it needs a bit more discussion of the biological/clinical implications.

Major suggestions:

1. I suggest a**dding more figures with actual cells from dataset referenced in [27] with perturbed masks overlaid to help readers see precisely why/where/when masks are not overlapping with specific markers**. This would add a lot more weight to the paper.

Thanks for this suggestion. We have added this accordingly to Fig S5 where we show the expression of selected markers as overlay with the ground truth and perturbed segmentations.

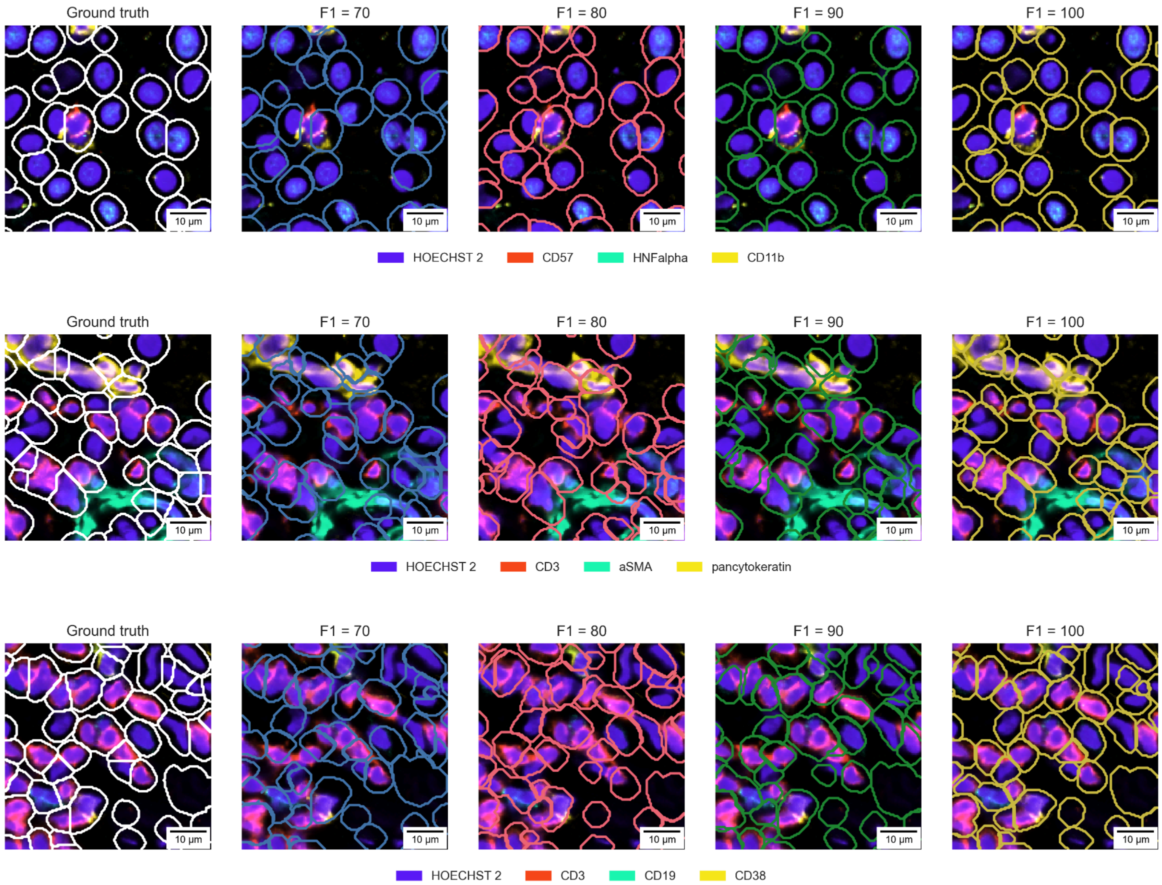


2. I would not have initially thought of using translation, rotation, affine, etc as ways to perturb masks. Segmentation errors are not caused by shifting the masks around like that. **A more intuitive transformation would be to do subtle warping, which many image analysis packages can do.**

We appreciate your insightful comment on perturbation methods. We agree that warping is an intuitive approach, and we did consider transformations like ElasticTransform (e.g., from Albumentations) during our development. For this study, we selected affine transformations primarily for two reasons:

1. Granular Control: They provided superior granular control, which was crucial for systematically generating the finely graded spectrum of error magnitudes central to our analysis of downstream impacts.
2. Interpretability & Parameterization: Their properties allowed for more direct parameterization and interpretation, facilitating controlled deviations from the reference masks.

3. Line 360: "potentially misleading biological conclusions": While this is true, it might be helpful to check with a clinician/biologist if this statement can be made more specific. We know that cell type calling can be inaccurate due to segmentation. How would that influence diagnosis of disease? Maybe the number of T cells infiltrating a tumor is l**ower measured leading to a wrong treatment outcome.**

We thank the reviewer for this comment on Line 360. We agree that the ramifications of inaccurate cell type calling can indeed be severe. For instance, as the reviewer rightly pointed out, if the number of tumor-infiltrating T cells is misquantified due to issues like segmentation errors, this could lead to flawed clinical diagnoses or inappropriate treatment decisions. We added this line to the respective text passage (line 391-396):

*A relevant real-world illustration can be seen with biomarkers such as the one developed by Salié et al. for predicting response to immune checkpoint inhibitor (ICI) therapy. This biomarker relies on accurate counts of CD8+ T cells, B cells, and CD4+ T cells. An incorrect measurement of these cell populations could directly result in patients who would be excellent candidates for ICI therapy being overlooked and thus denied a potentially effective treatment.*

**4. Line 368-370: This is a good idea but I've read many papers propose to model the segmentation error into their analysis but no one goes beyond to state more clearly how this would be done. What exactly would this look like? Please add 2-3 more sentences on how an error model could be used and introduced.**

We added a modification at line 406-410.

Minor revisions:
1. Since this is a segmentation-centric paper, **I suggest adding more references to the introduction on existing segmentation packages** used for high plex imaging such as Stardist, UnMICST, MIMO-NET, and MICRO-NET since they all have different strengths and weaknesses.

This has been done.

2. **Figure 1 shows an example of an original vs perturbed mask for an entire tissue. These panels look identical to each other because they are zoomed out.** A much more effective use of space is to show zoomed in versions where readers can actually see changes in mask shape. Also, scale bars should be added to all panels where applicable.

We thank the reviewer for raising this point. We adopted the suggestion and changed Figure 1 accordingly.

3. **Please state more clearly whether all transformations (translation, rotation, etc) are done in combination or just one.**

This has been done.

4. Line 160: you state that there are no comprehensive benchmarking dataset with ground truth. This is not true. **UnMICST has manually annotated outlines of nuclei from human tissue.**

Thank you for highlighting the UnMICST dataset. This dataset provides ground truth for nuclear outlines. Our statement in Line 160 was made in the context of our study's focus on full cell segmentations (including cytoplasm and membrane), which are essential for our specific downstream analyses. We have revised the corresponding line to clarify this distinction. The text now more precisely refers to the

availability of datasets with ground truth for full cell segmentations, while also acknowledging important resources like UnMICST for nuclear segmentation data.

5. Figure 2B: Suggest to overlay curves for all F1 scores for each marker in Figure 2B over each other for easier comparison.

We updated the figure accordingly.

6. Line 234: clarify if this is done on all cells in aggregate or specific cell type?
It is done on all cells. We rephrased this sentence to “This result becomes more obvious when considering the distribution of intersection-over-union values for all cell pairs”.

7. Fig. 3, 4, 5 : state how many cells/data points are represented by each bar?
We clarified this in the Results section in line 190-191.

8. Fig. 4 and 5: title "F1/preprocessing" is confusing. What does that mean?
This is the title of the legend. It is meant to explain the color coding for both perturbation level (70, 80, 90, 100) and applied preprocessing (log1p or None). To make sure this is clear to the reader we adapted the according figure captions.

9. Fig. 6B & S4: What do the colors mean? Add a color map.
We added “Color encodes the percentage of classified cells. For visual appearance, only off-diagonal elements are colored in.” to the according figures. We feel that a colormap is not needed since we added explicit values as text to the figure.

10. Line 317: "4% of CD T cells are incorrectly assigned". Is this a typo? This is not seen in matrix.
Thank you for your careful review and for spotting this error in Line 317. We appreciate your attention to detail. Yes, the original statement "4% of CD T cells are incorrectly assigned" was indeed a typo, and we apologize for this oversight. We have corrected this in the revised manuscript. The sentence in Line 343 now accurately states: "2% of CD4 T cells are incorrectly assigned to Tregs."

11. Please discuss why immune cells appear to be the most affected, according to 6B. Immune cells are smaller than tumor cells. Could this be why?

Thanks for this insightful question. We agree that the smaller size of immune cells is a primary reason for their heightened sensitivity to segmentation errors in Figure 6B. A given absolute error disproportionately impacts their area and derived features because they're represented by fewer pixels. We added a brief discussion to the manuscript clarifying this size-dependent effect (line 346-348).

12. You list CODEX and MIBI as examples of imaging modalities in the introduction. These have wildly different imaging resolutions. Please discuss in what way significant segmentation errors might be for different resolutions.
While we agree resolution is a key factor influencing such errors, a systematic comparison of resolution-specific error profiles across these distinct technologies is beyond our current study's scope. Our work focuses on the impact of general segmentation boundary errors, using CODEX as our model

system.

13. Fig S04: panels need more vertical spacing.
We added spacing for a better visual appearance.

14. Line 376: If interested, there are other perturbations one can do/discuss. Change resolution by downsizing, change bit depth from 16bit to 8bit, background subtraction.
Thank you for suggesting other perturbation types like changes to image resolution, bit depth, or background subtraction. Our current study specifically focuses on modeling perturbations that directly mimic errors arising from the cell segmentation process itself given a dataset with fixed resolution and bit depth and preprocessing. In this setting errors arise through inaccuracies in the output cell masks (e.g., incorrect boundary delineation). The alterations you suggested primarily affect input image quality or pre-processing steps. While these are certainly important factors for overall image analysis pipelines, they represent a different category of variations than the direct segmentation algorithm output errors that are the core subject of our current investigation. Therefore, for this study, we've concentrated on these mask-level perturbations.

15. Github landing page could use more information about running code.
The Github page was updated to provide information on installing the needed environment etc.

Reviewer #3: Bruhns et al. describe the downstream effect of using inaccurate cell segmentation results in single-cell analysis. Cell segmentation of a tissue is done to add spatial depth to a single-cell data by linking phenotype to their protein expression. However, the author shows that errors from many segmentation methods can have downstream effects especially in clustering by cell type classification and neighborhood mapping of the single-cell data. The authors took precaution to create "realistic" segmentation errors–called a perturbation mask–by a combination of 7 types of affine image transformation methods to mimic errors that may occur in segmentation using other more established methods. The authors also created and **enhanced a parameter to evaluate the degree of this perturbation when visually compared to the ground truth data called IoU-F1.** They investigate the accuracy and similarity of the neighborhood mapping and clustering using both Jaccard index and Adjusted Rand Index with different neighbors and F1 values. Finally, the authors used Gaussian Mixture Model-based phenotyping to measure how accurately the dataset segmented using different F1 values of perturbation classify the cell types of all cells in the liver dataset.

We thank the reviewer for the thorough and accurate summary of our work; we are pleased that the main aspects were clearly conveyed.

We would like to offer a slight clarification regarding the IoU-F1 score. While we indeed utilized the IoU-F1 score extensively - specifically to measure the discrepancy between our synthetically perturbed segmentations and the reference segmentations from Ruf et al. - this metric is a widely established community standard for evaluating segmentation accuracy (e.g., in segmentation benchmarks) and was not a novel contribution developed by us.

Our primary original contributions are: (1) the methodology for generating realistic in-silico segmentation perturbations that mimic common errors, and (2) the systematic analysis of how these controlled perturbations affect various downstream single-cell analyses.

Overall, this is a timely and informative study as spatial transcriptomics as a field is rapidly expanding, and this study takes a systematic approach to probe how downstream analysis can be affected due to a variety of segmentation errors. While the text was generally easy to follow (with certain exceptions, noted below), the figures need substantial work for readability and significantly below publication standards. Often appropriate statistics are missing thus diluting and questioning the conclusions made by the authors. We have several comments and concerns, in no particular order, that should be addressed:

We could not find Code or a README file. For a study that is benchmarking, providing the code and related data is indispensable to the review process.

We fully agree that providing the code is indispensable for reproducibility and the review process. The complete source code and an updated README file are available in our repository at: https://github.com/mbruhns/SegmentationErrorBenchmark

The README file, located in the root of the repository, has been revised to clearly outline the project structure and guide users. All analysis code can be found within the `/src` folder. We trust this addresses the reviewer's concern.

Unclear if the authors expect their work to be a “tool” for others to use or a conceptual point for others to keep in mind. We recommend the authors add this nuance to the introduction and discussion.

Thank you for this helpful feedback. We agree that this distinction was not made sufficiently clear in the original manuscript.

Our work is intended primarily as a conceptual contribution, highlighting important considerations and providing a framework for future analyses, rather than being presented as a ready-to-use software tool.

We have now explicitly clarified this perspective in the revised manuscript. As per your recommendation, we have incorporated this nuance into both the Introduction (line 34) and Discussion (lines 396-398, 414-416) sections.

For all figures: please add full forms of all acronyms used either in the figure or figure caption. Similarly, all labels should be adequately defined. As it stands, the figures presented a significant cognitive burden while evaluating this manuscript. Also add scale bars to all figures used.

We now define acronyms in captions.

**The argument for analyzing “phenotypes” (last two sections) is a bit weak as ground truth for cell-type labels are not possible with some markers. As such, this kind of analysis is no different from what is done in the rest of the paper, including the cluster assignment analysis.**

We thank the reviewer for this discussion and acknowledge the inherent challenges in phenotyping, particularly concerning definitive ground truth with certain marker sets.

We wish to clarify that our hierarchical gating analysis provides a distinct and complementary perspective to the presented unsupervised clustering analysis. While unsupervised methods are powerful for data-driven discovery of cell populations, our gating strategy is fundamentally knowledge-driven and

recapitulates the common strategy to assign single-cell cell types. It employs the well-characterized Ruf et al. marker panel to identify established, broader cell phenotypes according to the gating strategy described in the original study. This approach enhances the robustness of our interpretations, as focusing on these widely accepted categories helps mitigate potential ambiguities that can arise from marker limitations when defining highly granular or novel sub-populations.

Therefore, this targeted phenotyping is not redundant with our other clustering analyses. Instead, it uniquely enables us to specifically investigate and interpret biological changes (such as alterations in cell signaling) within these recognized cell types. This offers insights that are more directly translatable to existing biological frameworks and thereby complements the exploratory findings from unsupervised clustering.

The authors should consider supplementing figure S1 with real-data examples on how affine transformation changes a mask in addition to the squares.

We adapted Figure S1 accordingly and added an additional row showcasing the transformations on a mask.

For Figure 1, show pixel/cell to represent the pixel size as compared to the cell so that the one-pixel gap between cells can be created that is mentioned in both lines 54-56 and 147. We are curious how this would affect the error of the segmentation for cells that are very close to each other or overlap.

We analysed the adjacent cells for several of the reported settings. In no case we had more than 50 cells that were directly adjacent to each other. Since the order of magnitude of this is below 0.015% we argue that the effect of this correction to the overall segmentation error is negligible.

For Figure 1 again, there is a schematic of the pipeline created for this benchmark but showing a zoomed in picture of a group of cells (1-10) in brightfield and its masks would also help explain lines 149-154 to show how this "empirical selection process" selects a "realistic error". Furthermore, so we can visualize, F1 of the perturbation dataset in this figure can be stated.

We appreciate the reviewer's  suggestions for enhancing Figure 1. Our intention for this figure is to provide a clear, high-level schematic of the overall benchmarking pipeline without emphasis on actual quantitative benchmark results..

While adding detailed image crops or a specific F1 value could offer more granular context for an individual example, we believe this might overload Figure 1 and detract from its primary role as a conceptual overview. A single F1 score, for instance, could also be misconstrued as representing the entirety of our multi-level perturbation analysis.

To maintain Figure 1's clarity and focus as a concise schematic of the general workflow, we prefer to retain its current design. We are very grateful for the reviewer's constructive feedback.

Lines 118-125: Besides showing the equation and components of the equation, also explain how the "three components" mentioned in line 307 fit into this equation. What does the N values represent from the gating strategy that is shown in Figure S3?

We appreciate the reviewer's careful attention to detail and thank you for highlighting these points of ambiguity. We apologize for an error in formatting in the initial manuscript that made this section unclear.

In the revised manuscript, we have corrected the formatting to clearly distinguish between the introduction of Gaussian Mixture Models (GMMs) and the separate "Wu and Palmer" similarity metric. We believe this revision now properly contextualizes the equation presented and clarifies how its components (including the "three components" referred to from line 307, which pertain to the Wu & Palmer metric) are to be understood.

Regarding the "N" values and Figure S3: "N", as defined in the context of the Wu & Palmer metric, is not a parameter directly extracted from the visual details of the gating strategy shown in Figure S3. Rather, for the Wu & Palmer calculation, the hierarchical gating strategy itself is conceptualized as a phylogenetic tree. The "N" values then refer to measures of depth or path length within this tree structure, as part of the metric's computation. We have endeavored to make this distinction and the role of "N" clearer in the revised text.

Spell out or explain HALO from line 165. We think it warrants an explanation as to why this one in particular is used as compared to other segmentation tools.

Thank you for this important query. We have revised the manuscript to provide these clarifications. We now explicitly state that HALO is an image analysis platform developed by Indica Labs (line 182).

Regarding its use: our decision to utilize the HALO-generated segmentation as the ground truth was a practical one, based on adherence to the original peer-reviewed study from which this dataset was derived. Specifically, this segmentation was the reference standard provided and used in that source publication. We adopted this existing benchmark to ensure consistency with the established data, rather than undertaking a new segmentation or selecting HALO ourselves after a comparative evaluation of various tools.

We will ensure the manuscript clarifies this rationale and continue to acknowledge that this reference segmentation (like any segmentation) is understood to have its own potential imperfections, a context that is indeed relevant to our study on segmentation errors.

Figure 2A: Which one represents the original ground truth, an example with arrows or even a blow up version of one of the cells to show ground truth and perturbations? How did you compare the "visual appearance" "to determine noticeable artifacts"? Point out what is the small versus significant difference between the red-lined mask and the white-lined mask, assuming the white is reference. In general, this figure needs significant work for the reviewer to understand the key points.

We appreciate the reviewer's detailed feedback on Figure 2A and the suggestions for enhancing its clarity. To ensure the original ground truth and perturbed masks are clearly identifiable, we have revised the caption for Figure 2A. The updated caption now reads: "Comparison of the original (white) and perturbed cell masks (colored according to perturbation strength: blue, red, green, yellow) based on mask borders on a randomly selected image slide." This explicitly identifies the white-lined mask as the original ground truth and clarifies that the colored masks represent perturbations of varying strengths.

To better explain how "noticeable artifacts" were determined by visual appearance, we have expanded the relevant sentence in the manuscript. It now includes representative examples, such as: "...like obviously unrealistic cell shapes with respect to curvature and overall shape."

Figure 2A provides visual examples of deviations between the reference (white-lined) mask and various perturbed (colored) masks. The "small" versus “significant" nature of these differences across the entire dataset is quantitatively evaluated using the IoU-F1 score, as detailed in the manuscript. This score reflects that masks resulting from stronger perturbations (indicated by different colors in the figure, corresponding to different IoU-F1 score ranges) generally exhibit a greater deviation and decreased overlap when compared to the reference segmentation.

Figure 2D and Line 200. In the figure it shows as mean of absolute error of features while in the passage its stated as median. Which one are you referring to? Also, are there more explanations as to why Pearson correlation is used to calculate this correlation? This can be done in the Methods section.

Thank you for pointing us to the discrepant reference to reporting summary statistics like mean or median and the request for motivating the use of Pearson correlation as the correlation measure of choice . We appreciate the opportunity to clarify them.

1. Mean vs. Median (Figure 2D & Line 200): The reviewer is correct; "mean" is the intended and appropriate statistic here. We apologize for this inconsistency. We have revised the manuscript to ensure that both the legend for Figure 2D and the text in Line 200 consistently refer to the "mean absolute error of features."

2. Justification for Pearson Correlation: We agree that an explanation for using Pearson correlation is warranted. Pearson correlation was chosen because it is a standard and widely understood method for quantifying the linear relationship between two continuous variables, which directly applies to the association we are examining in this context.

Line 210: Please explain what the chance level is, is there a specific value for this threshold?

The term "chance level" refers to the baseline probability of a specific outcome occurring by chance. For a scenario with "N" equivalently likely outcomes or categories, the chance level is calculated as 1/N.

The specific numerical value for this threshold in the context of Line 210 would therefore be 1 divided by the number of relevant, equally probable outcomes (N) pertinent to that specific analysis. For example, in a classification task with 2 equally likely classes, the chance level would be 1/2, or approximately 0.5.

To make this more clear to the reader, we changed the wording to “the classifier can distinguish between original and perturbed data only slightly better than random guessing” (line 229)..

Line 233-234 shows a strong point to be made in the argument regarding the neighborhoods with distribution of IOU values which is proven with Figure S2. Placing it in Figure 3 would add importance to this information and be a stronger representation of the author's explanation.

We appreciate the reviewer's positive assessment of the point made in lines 233-234 and the supporting evidence in Figure S2. We also thank you for the suggestion to integrate Figure S2's content into Figure 3 to enhance its prominence.

Figure S2 is designed to illustrate the fundamental overlap properties of segmentations across various perturbation strengths. These characteristics are foundational and have direct implications for all subsequent downstream analyses presented in the manuscript, influencing the interpretation of not only Figure 3 but also other figures that follow.

To accurately reflect this broad relevance and to avoid inadvertently suggesting that the insights from Figure S2 pertain exclusively to the specific analysis shown in Figure 3, we believe maintaining Figure S2 as a distinct figure (or in its current supplementary location) is more appropriate. This ensures its general applicability to the various downstream effects we investigate is clear.

We hope this explanation clarifies our rationale for the current placement of this information.

Line 253-255: What does it mean to “consider the most similar cluster assignment for ground truth and perturbed data”? Are all the ARI represented only show the ones with the highest ARI value. It is never stated what is the range of the ARI value (typically -1 to 1 where 1 shows highest similarity) but the images only show ARI above 0. Do the authors calculate ARI with ranges 0-1? Furthermore, please elaborate on how this shows that the differences observed are only due to “introduced perturbations”. Could the authors provide quantitative evidence for their claim? Possibly a way to distinguish the noise between a clustering error and perturbation.

We thank the reviewer for these questions regarding our Adjusted Rand Index (ARI) calculations and presentation.

To clarify: yes, the ARI values represented in our analyses are indeed those corresponding to the most similar cluster assignment found between the ground truth and perturbed data. This procedure is detailed in the Methods section (lines 270-274 )and is implemented as such to ensure a conservative estimate of the impact of perturbations on clustering. Our aim is to avoid inflated discrepancy estimates that could arise from factors unrelated to the perturbations themselves, such as unfavorable clustering algorithm initialization.

Regarding the range of ARI values, we utilize the standard definition where the ARI can range from -1 (indicating disagreement less than expected by chance) to 1 (indicating perfect agreement). The figures display ARI values exclusively above 0 simply because our analyses with the current experimental data resulted in all ARI values being positive.

Figure 4 is only referred once in the text without mentioning the subfigures (4A, 4B, 4C and 4D). Reading Lines 258-272 creates confusion as to which subfigures each conclusion is pointed to.

We thank the reviewer for this comment, the manuscript has been updated accordingly (lines 279-285).

In each Figure 4 and Figure 5, subfigures A and C have the same caption. It is not shown or mentioned in the captions that there is a difference between the two is from application of log(x+1) transformation as a variance stabilization, nor which subfigure has variance stabilization or not. This "preprocessing variance" step mentioned in Line 259 creates confusion as it is not stated again what the difference is in that paragraph nor shown in the graph.

We thank the reviewer for pointing out that this specification in both figures was missing, we adapted accordingly. Line 259 mentions "preprocessing variants", not "preprocessing variance". It refers to the different ways of preprocessing applied in the experiments (no preprocessing and log1p).

Furthermore, Figure 5 A and C look identical, and Figure 5E does not have a label on its scale bar. How does the difference in color contribute to the claim in Line 291 that log(x+1) data affects clustering robustness? Please provide clear evidence to support this claim?

Thank you for your feedback on Figure 5 and Line 291. The visual similarity of Figures 5A and 5C simply reflects the highly comparable clustering outcomes these panels depict under their respective conditions. Regarding Figure 5E and its scale bar: we acknowledge your observation. As described in its caption, Figure 5E is a (2D) histogram, and the scale bar indicates the "count" of cells within each bin. Our claim in Line 291 that log(x+1) transformation affects clustering robustness is supported by Figure 5B in the submitted manuscript. This panel quantitatively compares clustering agreement with and without this transformation. While visual changes in related plots can be subtle, Figure 5B provides direct evidence of these effects, which, though modest, are consistently observed. We hope these clarifications of our submitted manuscript are helpful.

Overall when explaining differences between histograms of any metric no p-values are mentioned to show significance when comparisons are made. The comparisons by eye are of very little difference as also mentioned in Lines 292-294.

We appreciate the reviewer's observation regarding the presentation of statistical significance for histogram comparisons and the note on the visual subtlety of some differences, a point we also discuss in Lines 292-294 of the manuscript.

We deliberately chose not to report p-values. Our study is based on a simulation framework. A key characteristic of such simulation studies is the possibility to generate an arbitrary large number of simulated data items and thereby to adjust the statistical power to achieve statistical significance for arbitrarily small differences in distributions or effect sizes. Consequently, while statistical significance is an important tool for comparisons based on analysis of real data with an a priori fixed number of data items, p-values are of limited meaningfulness in our simulation study setting to discern the practical relevance or the systematic nature of observed effects.

Therefore, our analysis has placed a greater emphasis on the magnitude and consistency of changes in the distributions in response to the systematically introduced perturbations, rather than on reporting p-values for every comparison of histograms. The visual differences, even when subtle, are interpreted in the context of these controlled experimental manipulations, where consistent, directed shifts can be meaningful indicators of the perturbations' impact.

Lines 305-310: Please specify and elaborate on what are the "first two components" and the "three components" used to reduce the number of false-positive gates. It is mentioned that it's in reference to the diagram in Figure S3, a smaller and simpler diagram can be made to Figure 6 from Figure S3 that clearly shows what the components are and what cell type they represent or will positively gate.

Thank you for this question, which allows us to elaborate on our gating strategy and the role of mixture model components, as mentioned in Lines 305-310. We also appreciate the suggestion regarding a simplified diagram for Figure 6.

In our methodology, Gaussian Mixture Models (GMMs) are utilized to determine the positivity or negativity of individual markers for each cell. The "components" you refer to (e.g., the "first two components" or

"three components" in Lines 305-310) pertain to the number of Gaussian distributions fitted by the GMM to the expression values of a single marker.

- For instance, a two-component GMM for a given marker (e.g., CD3) would typically model two distinct populations: cells "negative" for CD3 and cells "positive" for CD3.
- A three-component GMM might be used to provide a more refined distinction, identifying "negative," "intermediate/low," and "high-positive" populations for that marker. By explicitly accounting for an intermediate or low-expressing group, the three-component model can help establish a more stringent and accurate threshold for defining the truly "positive" population. This improved thresholding is key to reducing false-positive gate assignments when determining a cell's status for that specific marker.

We have added the following sentence to lines 332-336 summarizing the above explanation.

*Once the positive/negative status for each relevant marker is determined using these GMMs, the overall gating strategy (conceptually illustrated in Figure S3) combines these individual marker statuses to classify cells into specific types. For example, a CD8 T cell would be defined by a combination such as CD45 positive, CD3 positive, and CD8 positive.*

Lines 310-312: Please elaborate or use equations to represent what balance accuracy meant in this context and the range of values. Would 0.8 be a representation of -1 to 1 or 0 to1 or 0 to 2?

We appreciate the reviewer's request for clarification regarding "balanced accuracy" as mentioned in Lines 310-312.

Balanced accuracy is a performance metric particularly useful for classification tasks with imbalanced datasets. In the context of binary classification, it is defined as the arithmetic mean of sensitivity (True Positive Rate, TPR) and specificity (True Negative Rate, TNR). For a multi-class scenario, it is generally calculated as the average of the recall (sensitivity) obtained on each class. The range of values for balanced accuracy is [0, 1]. We updated the methods section accordingly (lines 135-140).

Therefore, a balanced accuracy value of 0.8, as posed in your question, would represent an 80% average correct classification rate when accounting for potential imbalances between the classes.

Figure S3: The caption only indicates what extra information is added to the figure but does not show where cell lineage or cell subtype that is mentioned in Lines 314 and 317-320 refer to. Please write an elaborate description on the gating and how the categories mentioned in the lines above are classified through the diagram.

Thank you for your question regarding Figure S3 and how it depicts the cell lineages and subtypes referenced in Lines 314 and 317-320.

Figure S3 presents the flowchart of our gating strategy. To clarify its interpretation: the diagram is structured hierarchically. It begins with broader cell populations, and each subsequent gate, defined by the specific markers labeled on the flowchart, progressively refines these populations. "Cell lineages" are generally established by early gating decisions based on key lineage markers. The "cell subtypes" mentioned in the text are then identified through subsequent combinations of specific markers within these established lineage branches. Therefore, by tracing the path from the initial population through the sequence of marker-based gates to any given endpoint, one can determine the classification criteria and hierarchical origin (lineage and subtype) for that cell population as discussed in the manuscript.

We hope this explanation of how to read the existing Figure S3 in conjunction with its caption and the main text is helpful.

Line 320: “we use Wu and Palmer distance (see)” there seems to be missing information in this sentence. The Wu and Palmer distance is not explained nor mentioned in the Methods section to show how it quantifies the different errors.

We fixed the reference and adapted the methods section for clarification (line 124-133).

Line 286-287: Mentioned that with lower neighbors it has higher ARI values, which is not what is represented in Figure 5D. This also affects Line 289 as Figure 4D and Figure 5D have opposite trends, which is opposite of the claim that shows the result matches with k-means algorithm. Figure 5D the differences between each bar looks very small with no significance. Higher number of neighbors induces fewer clusters can be seen in Figure 5E but not from 5D.

Thank you for identifying the error in Lines 286-287 regarding Figure 5D's trend. We have corrected this: any ARI trend in Figure 5D with varying neighbors is indeed minor, if not opposite to our initial statement, and the text now accurately reflects this (lines 306-308):

*The number of neighbors has negligible effect on clustering agreement, at least for values that fall in the typical range for typical single-cell clustering analyses from multiplexed tissue imaging data (Fig 5 D).*

Regarding the comparison between k-means (Figure 4D) and Leiden (Figure 5D) and Line 289: The critical difference lies in the parameters. "k" in k-means directly sets cluster numbers. Conversely, Leiden's "number of neighbors" typically has an inverse impact on the final cluster count, an effect clearly shown in Figure 5E. Consequently, direct ARI trend comparisons based on increasing these distinct parameters can be misleading.

Line 288: Please elaborate and show evidence on the claim that neighborhood size "amplifies sensitivity" to perturbation-induced feature shifts.

Upon review, we acknowledge that our current manuscript's data do not strongly demonstrate this specific amplification effect within the tested range of neighborhood sizes. The statement was based on the expectation that such effects might become more pronounced at extreme parameter values, given how neighborhood size can influence cluster formation. However, we concede this particular dynamic is not clearly evidenced by our present results. We appreciate you prompting this clarification and adapted the manuscript accordingly (lines 306-310).

Why have the authors not considered using one or two other segmentation tools on the same dataset and no perturbations on the metric as well?

We appreciate the reviewer's questions, which allow us to clarify the specific scope and methodology of our study.

1. Regarding the suggestion to use other segmentation tools on the same dataset:

Our primary research goal in this manuscript is to systematically investigate the impact of varying degrees and types of segmentation errors on downstream single-cell analyses. To achieve this in a controlled manner, we apply defined perturbations to a reference segmentation and then analyze the consequences. While a comparative study benchmarking the performance of different segmentation tools

on the dataset would indeed be valuable, it represents a distinct research question focused on tool evaluation rather than our current aim of understanding the generalizable effects of segmentation inaccuracies with respect to their strength.

2. Regarding the query about "perturbations on the metric":

In our experimental design, the perturbations are intentionally applied to the cell segmentations to simulate a range of common errors. The metrics we employ (e.g., IoU-F1 for segmentation quality, ARI for clustering agreement) are then used as stable, objective instruments to:

A. Quantify the magnitude of the introduced perturbations in the segmentations (by comparing perturbed masks to the reference).
B. Evaluate the effect of these perturbed segmentations on the outcomes of subsequent downstream analyses.

The metric itself is not subjected to perturbation; it serves as a consistent yardstick. Altering or "perturbing" the evaluation metric would undermine our ability to reliably measure and interpret the impact of the segmentation errors, which are the actual focus of our investigation.

We hope this explanation clarifies the rationale behind our current study design and methodological choices.

A brief discussion on why CODEX was primarily chosen for analysis could be beneficial for the study and how their approach extends to other modalities and technologies.

We appreciate the reviewer's suggestion to elaborate on our choice of the CODEX dataset and the broader applicability of our approach. We have expanded on these points in the Discussion section of the revised manuscript.

1. Rationale for Using the CODEX Dataset:

The CODEX dataset was selected for this study primarily due to its public availability and comprehensive nature, which made it highly suitable for our initial proof-of-concept investigation. Its characteristics allowed us to rigorously develop and demonstrate our perturbation analysis framework, focusing on the impact of simulated segmentation errors rather than on dataset-specific preprocessing idiosyncrasies.

2. Extensibility to Other Modalities and Technologies:

While the current work is implemented using CODEX data, the fundamental principles of our perturbation analysis are designed to be broadly applicable. The challenge of cell segmentation and the potential for errors to propagate into downstream analyses are common across various highly multiplexed tissue imaging modalities (e.g., MACSima, CyCIF, MIBI, Imaging Mass Cytometry) and other image-based cytometry technologies. Therefore, we anticipate that our approach to simulating segmentation errors and evaluating their impact can be readily extended to these other platforms. As we state in the Discussion: "This study is based on a single dataset and several perturbations thereof. It serves as a proof-of-concept to showcase the underlying issue. Future research will expand on performing this perturbation analysis for further datasets." We have now further elaborated on this point to explicitly include the expected transferability to different technologies.

In general, the authors are strongly recommended to expand their metrics for calculating errors and

benchmarking for different modalities/aspects. In the single-cell RNA sequencing space, this has been thoroughly investigated for singlet/doublet detection by the following papers: PMID: 33338399 and PMID: 38925122. Perhaps a discussion on how the downstream interpretations are often affected and propagated in the field of scRNAseq (which was a precursor to modern spatial biology) through these mentioned papers and lessons learnt could be informative for inspiring the present study.

We thank the reviewer for their suggestion to draw parallels with the scRNA-seq field and for providing the references concerning error propagation, such as in doublet detection.

Thank you for your insightful suggestion regarding scRNA-seq parallels. We agree that an analogy exists for doublets. “Merged cells” in our imaging data, where multiple cells are incorrectly segmented as one is conceptually similar to scRNA-seq doublets. Such artifacts can indeed spur misleading interpretations, like incorrect cell type identification. Our current methodology, as applied in the submitted manuscript, already incorporates steps to mitigate these issues. We filter segmented objects based on mask area to exclude those that are unrealistically large (potential 'doublets') or too small. Furthermore, we apply quality control using nuclear marker intensity to remove cells with aberrant signals, which also helps identify and exclude problematic segmentations indicative of merged cells. These existing strategies in our work address concerns analogous to those in the scRNA-seq domain by aiming to control for major segmentation artifact types. We hope this clarifies our current approach.

Furthermore, regarding metrics for calculating errors and benchmarking, we find that the approaches used in the cited scRNA-seq benchmarking studies are conceptually well-aligned with our own. For instance, Xi et al. (PMID: 33338399) leverage precision, recall, and true negative rate to evaluate methods. Our study primarily uses the F1 score, which, as the harmonic mean of precision and recall, captures similar essential aspects of performance in evaluating segmentation accuracy.

Reviewer #4: This paper employs a systematic perturbation framework to simulate realistic segmentation errors, using affine transformations and binary opening, and evaluate their effects on key tasks such as k-nearest neighbor (kNN) graph construction and unsupervised clustering (k-Means and Leiden). The study is timely and relevant. However, there are several areas need clarification:

1. The Results sections often present trends (e.g., ARI decrease with perturbation strength, Fig 4 and 5; balanced accuracy below 0.8, Fig 6A) without providing specific numerical values or statistical tests to confirm significance.

We deliberately chose not to report p-values. Our study is based on a simulation framework. A key characteristic of such simulation studies is the possibility to generate an arbitrary large number of simulated data items and thereby to adjust the statistical power to achieve statistical significance for arbitrarily small differences in distributions or effect sizes. Consequently, while statistical significance is an important tool for comparisons based on analysis of real data with an a priori fixed number of data items, p-values are of limited meaningfulness in our simulation study setting to discern the practical relevance or the systematic nature of observed effects.

Therefore, our analysis has placed a greater emphasis on the magnitude and consistency of changes in the distributions in response to the systematically introduced perturbations, rather than on reporting p-values for every comparison of histograms.

2. Several methodological details are missing or unclear, limiting the reproducibility. For example, the range of k values for k-Means and Leiden clustering is not specified.

We appreciate the reviewer's feedback regarding the clarity of methodological details and the importance of reproducibility. We have addressed the specific query about clustering parameters as follows:

1. k-Means Clustering: You are correct that the range of "k" values (number of clusters) used for k-Means clustering needs to be explicitly stated for reproducibility. While Figure 4 illustrates the outcomes for the various "k" values we tested, we have now also explicitly specified this range (e.g., "k was varied from X to Y in steps of Z") in the Methods section under the subsection describing k-Means clustering.

2. Leiden Clustering: For the Leiden algorithm, "k" (as a predefined number of clusters) is not a direct input parameter. Instead, the Leiden method identifies communities by optimizing a modularity, and the number of resulting clusters is influenced by other parameters. In our study, these include the number of neighbors used in the construction of the k-Nearest Neighbors (KNN) graph underlying the clustering. We have reviewed the manuscript to ensure that all key parameters for the methods used are clearly described to support the reproducibility of our work. Figure 5E furthermore maps the considered number of neighbors for Leiden analysis with the resulting number of clusters.

3. The authors rely on visual inspection (Fig 2A) to determine that the perturbed masks maintain a consistent structure with the original masks, with "no apparent irregularities or distortions." However, visual inspection is inherently subjective and lacks quantitative rigor.

We appreciate the reviewer's comment regarding visual inspection and agree that quantitative rigor is paramount.

Our study primarily employs diverse quantitative metrics (such as protein expression correlation, cell area and IoU-based F1 scores amongst others) to rigorously assess the impact of perturbations; these form the core of our evaluation.

Visual inspection, as referenced for Figure 2A, serves as a complementary qualitative check. Its purpose is to confirm that our in-silico perturbation method generates structurally plausible (though intentionally erroneous) masks and does not introduce gross, unintended artifacts (e.g non-cell-like fragmentation or unrealistic shapes). This helps identify complex aberrations potentially missed by global quantitative measures.

4. The authors interpret the classifier's low accuracy as evidence of realistic perturbations due to high similarity between original and perturbed data, but, this interpretation may be confounded by alternative explanations, such as the classifier being underpowered or the selected features. I recommend discussing these alternative explanations for the classifier's performance.

We thank the reviewer for highlighting the need to discuss alternative explanations for our classifier's performance. We would like to highlight the context in which we apply the classifier 2-sample test, i.e. that we dispose over approximately 200,000 samples per class, where it is expected that most established classifiers will achieve their optimal classification performance.

Specifically, we employed CatBoost, a high-performance gradient boosting algorithm renowned for its robustness and effectiveness (e.g., Florek and Zagdański, 2023), chosen specifically to minimize concerns that an underpowered model might explain the results. Our interpretation is as follows: if an

effective classifier like CatBoost, when applied to relevant features, still achieves low accuracy in distinguishing the original data from the perturbed data, this suggests a high degree of similarity between these datasets. This high similarity, in turn, supports our conclusion that the perturbations generated are subtle and realistic.

We enhanced our C2ST framework by integrating a fully connected neural network as the core classifier, replacing CatBoost. This network, featuring two hidden layers of 360 neurons each and ReLU activation, learned the same classification task from identical features. With nearly 150,000 trainable parameters and substantial training data (approximately 200,000 samples per class), this setup provides suitable capacity for robust performance. Still, the classifier was not able to distinguish between the original perturbed data.

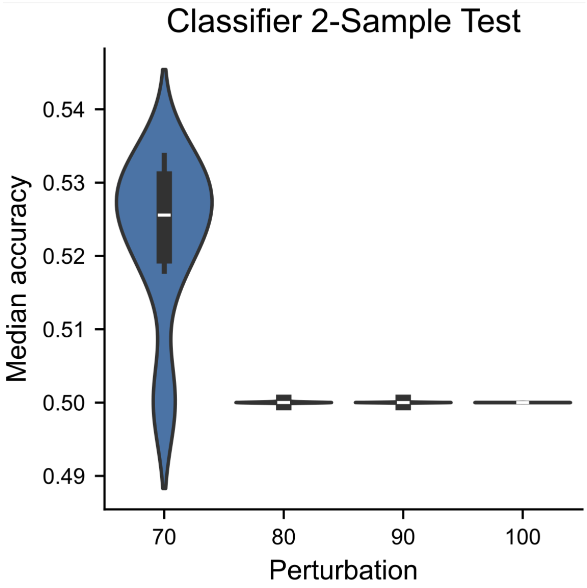

Supplement: Attachment — Submitted filename: Response to Reviewers.pdf [file pcbi.1013350.s008.pdf]
